# *N*′-[(*E*)-2-Hy­droxy-5-iodo­benzyl­idene]-4-methyl­benzene­sulfono­hydrazide

**DOI:** 10.1107/S1600536812035738

**Published:** 2012-08-23

**Authors:** Massomeh Ghorbanloo, Behrouz Notash

**Affiliations:** a Department of Chemistry, Faculty of Science, University of Zanjan, 45371-38791 Zanjan, Iran; b Department of Chemistry, Shahid Beheshti University, G. C., Evin, Tehran 1983963113, Iran

## Abstract

In the title mol­ecule, C_14_H_13_IN_2_O_3_S, the dihedral angle between the planes of the benzene and toluene rings is 84.3 (3)°. The mol­ecule displays a *trans* conformation with respect to the C=N bond. There is an intra­molecular O—H⋯N hydrogen bond with the azomethine N atom as acceptor. In the crystal, N—H⋯O hydrogen bonds connect the mol­ecules into chains running along the *b* axis.

## Related literature

For background to sulfonamides, see: Kayser *et al.* (2004 ▶). For related structures and their applications, see: Shahverdizadeh *et al.* (2011 ▶); Ali *et al.* (2007 ▶); Tierney *et al.* (2006 ▶); Silva *et al.* (2006 ▶). For polymorphism in sulfono­hydrazides, see: Kia *et al.* (2008 ▶); Tai *et al.* (2009 ▶).
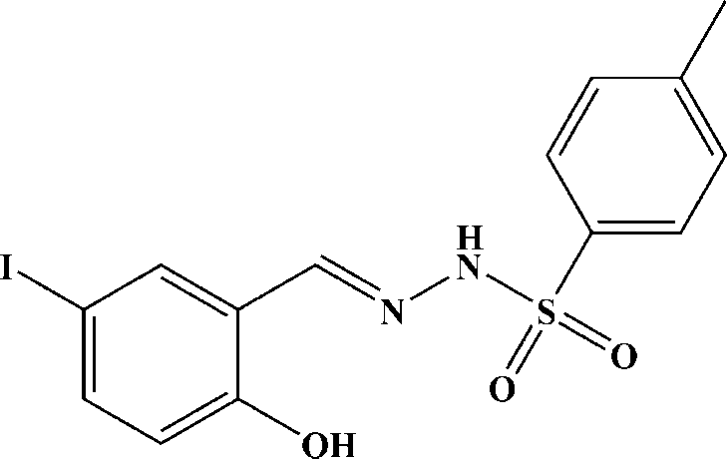


## Experimental

### 

#### Crystal data

C_14_H_13_IN_2_O_3_S
*M* *_r_* = 416.23
Monoclinic, 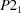

*a* = 6.2467 (12) Å
*b* = 10.394 (2) Å
*c* = 11.971 (2) Å
β = 92.42 (3)°
*V* = 776.6 (3) Å^3^
*Z* = 2
Mo *K*α radiation
μ = 2.21 mm^−1^
*T* = 298 K
0.50 × 0.40 × 0.20 mm

#### Data collection

Stoe IPDS 2 diffractometer
Absorption correction: numerical (*X-SHAPE*; Stoe & Cie, 2005 ▶) *T*_min_ = 0.405, *T*_max_ = 0.667
6035 measured reflections
3841 independent reflections
2791 reflections with *I* > 2σ(*I*)
*R*_int_ = 0.059

#### Refinement

*R*[*F* ^2^ > 2σ(*F* ^2^)] = 0.046
*wR*(*F* ^2^) = 0.114
*S* = 0.92
3841 reflections
197 parameters
3 restraints
H atoms treated by a mixture of independent and constrained refinement
Δρ_max_ = 0.88 e Å^−3^
Δρ_min_ = −0.99 e Å^−3^
Absolute structure: Flack (1983 ▶), 1641 Friedel pairs
Flack parameter: −0.06 (3)

### 

Data collection: *X-AREA* (Stoe & Cie, 2005 ▶); cell refinement: *X-AREA*; data reduction: *X-AREA*; program(s) used to solve structure: *SHELXS97* (Sheldrick, 2008 ▶); program(s) used to refine structure: *SHELXL97* (Sheldrick, 2008 ▶); molecular graphics: *ORTEP-3 for Windows* (Farrugia, 1997 ▶); software used to prepare material for publication: *WinGX* (Farrugia, 1999 ▶).

## Supplementary Material

Crystal structure: contains datablock(s) I, global. DOI: 10.1107/S1600536812035738/bt5993sup1.cif

Structure factors: contains datablock(s) I. DOI: 10.1107/S1600536812035738/bt5993Isup2.hkl

Supplementary material file. DOI: 10.1107/S1600536812035738/bt5993Isup3.cml

Additional supplementary materials:  crystallographic information; 3D view; checkCIF report

## Figures and Tables

**Table 1:** Hydrogen-bond geometry (Å, °)

*D*—H⋯*A*	*D*—H	H⋯*A*	*D*⋯*A*	*D*—H⋯*A*
O1—H1⋯N1	0.82 (2)	1.91 (5)	2.589 (6)	139 (7)
N2—H2*A*⋯O1^i^	0.86 (2)	2.05 (2)	2.914 (6)	173 (6)

Symmetry code: (i) 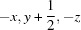.

**Fig. 1.**
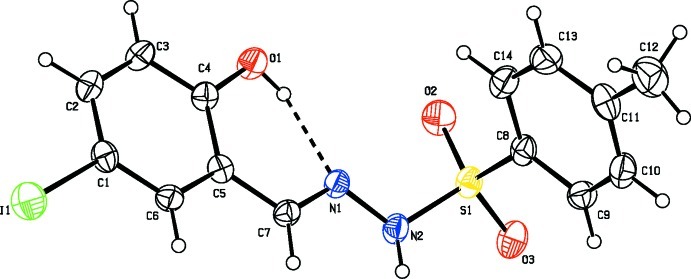
The molecular structure of the title compound with atom labels. Anisotropic displacement ellipsoids drawn at 30% probability level for non-H atoms.

**Fig. 2.**
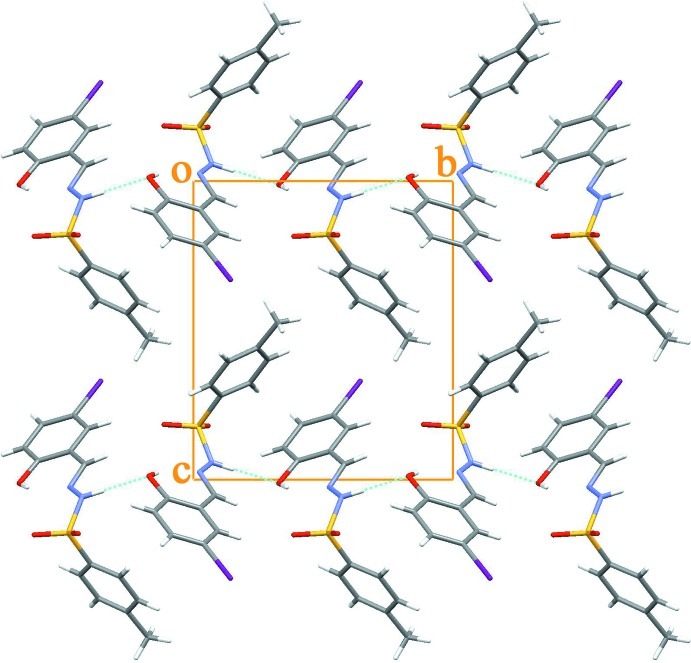
The packing diagram of the title compound. Hydrogen bonds are shown as blue dashed line.

## supplementary crystallographic information

### Comment

Sulfonyl hydrazones are found to exhibit large medicinal applications. Similar to sulfonamides, sulfonyl hydrazones also have various biological activities (Kayser *et al.*, 2004). For example, imidosulfonylhydrazones have antibacterial and antineociceptive properties (Silva *et al.*, 2006). Acidic sulfonyl hydrazone derivatives have analgesic and anti-inflammatory activities. On the other hand, polymorphism is a phenomenon wherein the same substances exhibits different crystal packing arrangements and is of practical importance *e.g.*, pharmaceutical processes where different physical properties of polymorphic forms can substantially alter the viability and quality of product. Plymorphism is another interesting subject in sulfonyl hydrazones. sulfonyl hydrazones derived from the condensation of *O*-hydroxy aldehydes and sulfonyl acid hydrazides can form different polymorphs. Kia *et al.* (2008) and Tai *et al.* (2009) have reported two polymorph of these type of compounds.

We report here the crystal structure of (*E*)-*N*'-(2-hydroxy-5-iodobenzylidene)-4-methylbenzenesulfonohydrazide. The asymmetric unit of the title compound contains one molecule, which is shown in Fig. 1. Bond distances and bond angles are in the normal range of similar compounds (Shahverdizadeh *et al.*, 2011; Ali *et al.*, 2007; Tierney *et al.*, 2006). The molecule displays *trans* configuration with respect to the C=N bond. The packing diagram of the title compound is shown in Fig. 2. In the title compound, the dihedral angle between the planes of benzene and toluene rings is 84.3 (3)°. There is an intramolecular O—H···N hydrogen bond in which the nitrogen of the azomethine group (–C=N–) acting as hydrogen bond acceptor. Intermolecular N—H···O hydrogen bond stabilize the crystal structure (Fig. 2 & Table 1).

### Experimental

For preparing the title compound, a methanol (10 ml) solution of 2-hydroxy-5-iodobenzaldehyde (2 mmol) was dropwise added to a methanol solution (10 ml) of 4-methyl-benzenesulfonic acid hydrazide (2 mmol), and the mixture was refluxed for 3 hrs. Then the solution was evaporated on a steam bath to 5 ml and cooled to room temperature. A white precipitate of the title compound was separated and filtered off, washed with 5 ml of cooled methanol and then dried in air. X-ray quality crystals of the title compound were obtained from methanol by slow solvent evaporation. Yield: 90%. Selected IR (cm^-1^): 3464 (w, broad), 3140 (*m*), 1619 (*s*), 1481 (*vs*), 1359 (*s*), 1329 (*vs*), 1264 (*vs*), 1177 (*s*), 1087 (*s*), 956 (*vs*), 869 (*vs*) 772 (*s*), 666 (*s*), 545 (*s*), 458 (*m*).

### Refinement

The hydrogen atoms bonded to O and N atoms were found in difference Fourier map and there coordinates were refined with *U*iso(H) = 1.2 *U*eq(O,N). The O—H and N—H distances were restrained to 0.82 (2)Å and 0.86 (2)Å, respectively. H atoms bonded to C were positioned geometrically and refined as riding atoms with C—H = 0.96 Å and *U*iso(H) = 1.5 *U*eq(C) for the methyl group and C—H = 0.93 Å and *U*iso(H) = 1.2 *U*_eq_(C) for the other H atoms.

### Crystal data

**Table:**

C_14_H_13_IN_2_O_3_S	*F*(000) = 408
*M**_r_* = 416.23	*D*_x_ = 1.780 Mg m^−^^3^
Monoclinic, *P*2_1_	Mo *K*α radiation, λ = 0.71073 Å
Hall symbol: P 2yb	Cell parameters from 3841 reflections
*a* = 6.2467 (12) Å	θ = 1.7–29.2°
*b* = 10.394 (2) Å	µ = 2.21 mm^−^^1^
*c* = 11.971 (2) Å	*T* = 298 K
β = 92.42 (3)°	Plate, colorless
*V* = 776.6 (3) Å^3^	0.50 × 0.40 × 0.20 mm
*Z* = 2	

### Data collection

**Table:**

Stoe IPDS 2 diffractometer	3841 independent reflections
Radiation source: fine-focus sealed tube	2791 reflections with *I* > 2σ(*I*)
Graphite monochromator	*R*_int_ = 0.059
Detector resolution: 0.15 mm pixels mm^-1^	θ_max_ = 29.2°, θ_min_ = 1.7°
rotation method scans	*h* = −7→8
Absorption correction: numerical (*X-SHAPE*; Stoe & Cie, 2005)	*k* = −14→13
*T*_min_ = 0.405, *T*_max_ = 0.667	*l* = −16→16
6035 measured reflections	

### Refinement

**Table:**

Refinement on *F*^2^	Secondary atom site location: difference Fourier map
Least-squares matrix: full	Hydrogen site location: inferred from neighbouring sites
*R*[*F*^2^ > 2σ(*F*^2^)] = 0.046	H atoms treated by a mixture of independent and constrained refinement
*wR*(*F*^2^) = 0.114	*w* = 1/[σ^2^(*F*_o_^2^) + (0.071*P*)^2^] where *P* = (*F*_o_^2^ + 2*F*_c_^2^)/3
*S* = 0.92	(Δ/σ)_max_ < 0.001
3841 reflections	Δρ_max_ = 0.88 e Å^−^^3^
197 parameters	Δρ_min_ = −0.99 e Å^−^^3^
3 restraints	Absolute structure: Flack (1983), 1641 Friedel pairs
Primary atom site location: structure-invariant direct methods	Flack parameter: −0.06 (3)

### Special details

**Table:**

Experimental. shape of crystal determined optically
Geometry. All e.s.d.'s (except the e.s.d. in the dihedral angle between two l.s. planes) are estimated using the full covariance matrix. The cell e.s.d.'s are taken into account individually in the estimation of e.s.d.'s in distances, angles and torsion angles; correlations between e.s.d.'s in cell parameters are only used when they are defined by crystal symmetry. An approximate (isotropic) treatment of cell e.s.d.'s is used for estimating e.s.d.'s involving l.s. planes.
Refinement. Refinement of *F*^2^ against ALL reflections. The weighted *R*-factor *wR* and goodness of fit *S* are based on *F*^2^, conventional *R*-factors *R* are based on *F*, with *F* set to zero for negative *F*^2^. The threshold expression of *F*^2^ > σ(*F*^2^) is used only for calculating *R*-factors(gt) *etc*. and is not relevant to the choice of reflections for refinement. *R*-factors based on *F*^2^ are statistically about twice as large as those based on *F*, and *R*- factors based on ALL data will be even larger.

### Fractional atomic coordinates and isotropic or equivalent isotropic displacement parameters (Å^2^)

**Table:**

	*x*	*y*	*z*	*U*_iso_*/*U*_eq_	
I1	0.80088 (6)	0.64270 (4)	−0.32973 (4)	0.07048 (17)	
S1	−0.2369 (2)	0.51804 (13)	0.17826 (10)	0.0423 (3)	
O1	0.2908 (7)	0.3287 (4)	0.0117 (4)	0.0493 (9)	
O2	−0.1752 (7)	0.3869 (4)	0.1850 (4)	0.0584 (11)	
O3	−0.4564 (6)	0.5561 (4)	0.1806 (4)	0.0566 (10)	
N1	0.0354 (7)	0.5237 (4)	0.0251 (3)	0.0387 (9)	
N2	−0.1570 (7)	0.5714 (4)	0.0580 (4)	0.0427 (10)	
C1	0.6247 (8)	0.5391 (5)	−0.2153 (4)	0.0437 (12)	
C2	0.6906 (8)	0.4168 (6)	−0.1848 (4)	0.0452 (12)	
H2	0.8103	0.3806	−0.2161	0.054*	
C3	0.5781 (9)	0.3485 (5)	−0.1074 (5)	0.0455 (12)	
H3	0.6245	0.2672	−0.0849	0.055*	
C4	0.3963 (8)	0.4012 (5)	−0.0633 (4)	0.0377 (10)	
C5	0.3257 (8)	0.5256 (5)	−0.0950 (4)	0.0369 (10)	
C6	0.4431 (8)	0.5934 (5)	−0.1716 (4)	0.0406 (11)	
H6	0.3999	0.6755	−0.1937	0.049*	
C7	0.1345 (8)	0.5822 (5)	−0.0518 (4)	0.0374 (10)	
H7	0.0837	0.6605	−0.0795	0.045*	
C8	−0.0891 (8)	0.6054 (5)	0.2828 (4)	0.0420 (12)	
C9	−0.1739 (10)	0.7173 (5)	0.3242 (5)	0.0456 (12)	
H9	−0.3088	0.7457	0.2992	0.055*	
C10	−0.0552 (11)	0.7865 (6)	0.4035 (5)	0.0543 (15)	
H10	−0.1116	0.8620	0.4317	0.065*	
C11	0.1458 (11)	0.7461 (6)	0.4418 (5)	0.0543 (14)	
C12	0.2764 (15)	0.8255 (10)	0.5268 (7)	0.082 (2)	
H12A	0.2910	0.7791	0.5960	0.123*	
H12B	0.2049	0.9058	0.5390	0.123*	
H12C	0.4158	0.8419	0.4991	0.123*	
C13	0.2264 (9)	0.6346 (9)	0.3970 (5)	0.0598 (14)	
H13	0.3618	0.6065	0.4214	0.072*	
C14	0.1134 (10)	0.5641 (6)	0.3177 (5)	0.0532 (14)	
H14	0.1717	0.4899	0.2880	0.064*	
H1	0.173 (6)	0.359 (7)	0.024 (6)	0.064*	
H2A	−0.186 (11)	0.649 (3)	0.037 (6)	0.064*	

### Atomic displacement parameters (Å^2^)

**Table:**

	*U*^11^	*U*^22^	*U*^33^	*U*^12^	*U*^13^	*U*^23^
I1	0.0614 (2)	0.0783 (3)	0.0737 (3)	−0.0046 (3)	0.02653 (18)	0.0203 (3)
S1	0.0421 (7)	0.0388 (6)	0.0469 (6)	−0.0040 (5)	0.0134 (5)	−0.0008 (5)
O1	0.057 (2)	0.0328 (19)	0.060 (2)	0.0003 (17)	0.019 (2)	0.0067 (16)
O2	0.070 (3)	0.042 (2)	0.064 (3)	−0.007 (2)	0.012 (2)	0.0030 (18)
O3	0.040 (2)	0.063 (2)	0.068 (2)	−0.0066 (18)	0.0153 (18)	−0.008 (2)
N1	0.041 (2)	0.036 (2)	0.040 (2)	0.0023 (18)	0.0073 (17)	−0.0028 (17)
N2	0.044 (2)	0.041 (2)	0.044 (2)	0.002 (2)	0.0133 (19)	−0.0004 (19)
C1	0.042 (3)	0.050 (3)	0.039 (2)	−0.008 (2)	0.008 (2)	0.001 (2)
C2	0.038 (3)	0.047 (3)	0.050 (3)	0.002 (2)	0.007 (2)	−0.008 (2)
C3	0.044 (3)	0.037 (3)	0.056 (3)	0.004 (2)	0.007 (2)	−0.004 (2)
C4	0.042 (3)	0.030 (2)	0.041 (2)	−0.005 (2)	0.005 (2)	−0.0026 (19)
C5	0.038 (2)	0.037 (3)	0.035 (2)	−0.006 (2)	0.0018 (19)	−0.002 (2)
C6	0.045 (3)	0.035 (2)	0.042 (2)	0.002 (2)	0.005 (2)	0.0055 (19)
C7	0.043 (3)	0.029 (2)	0.041 (2)	0.001 (2)	0.004 (2)	−0.0008 (18)
C8	0.039 (3)	0.044 (3)	0.044 (2)	−0.004 (2)	0.015 (2)	0.0020 (19)
C9	0.046 (3)	0.043 (3)	0.048 (3)	0.004 (2)	0.005 (2)	0.004 (2)
C10	0.067 (4)	0.047 (3)	0.051 (3)	0.002 (3)	0.018 (3)	−0.007 (2)
C11	0.060 (4)	0.064 (4)	0.040 (3)	−0.014 (3)	0.011 (2)	0.001 (3)
C12	0.086 (6)	0.099 (6)	0.059 (4)	0.000 (5)	−0.008 (4)	−0.013 (4)
C13	0.047 (3)	0.077 (4)	0.055 (3)	0.011 (4)	0.005 (2)	0.011 (4)
C14	0.050 (3)	0.052 (3)	0.059 (3)	0.007 (3)	0.013 (3)	−0.003 (3)

### Geometric parameters (Å, º)

**Table:**

I1—C1	2.092 (5)	C5—C7	1.447 (7)
S1—O2	1.418 (5)	C6—H6	0.9300
S1—O3	1.429 (4)	C7—H7	0.9300
S1—N2	1.640 (4)	C8—C9	1.379 (7)
S1—C8	1.774 (5)	C8—C14	1.384 (8)
O1—C4	1.364 (6)	C9—C10	1.381 (9)
O1—H1	0.82 (2)	C9—H9	0.9300
N1—C7	1.283 (6)	C10—C11	1.384 (9)
N1—N2	1.373 (6)	C10—H10	0.9300
N2—H2A	0.86 (2)	C11—C13	1.380 (11)
C1—C2	1.380 (8)	C11—C12	1.521 (11)
C1—C6	1.389 (7)	C12—H12A	0.9600
C2—C3	1.382 (8)	C12—H12B	0.9600
C2—H2	0.9300	C12—H12C	0.9600
C3—C4	1.385 (7)	C13—C14	1.371 (10)
C3—H3	0.9300	C13—H13	0.9300
C4—C5	1.413 (7)	C14—H14	0.9300
C5—C6	1.390 (6)		
			
O2—S1—O3	121.6 (3)	C5—C6—H6	119.9
O2—S1—N2	106.4 (2)	N1—C7—C5	119.6 (4)
O3—S1—N2	104.6 (3)	N1—C7—H7	120.2
O2—S1—C8	108.7 (3)	C5—C7—H7	120.2
O3—S1—C8	108.4 (2)	C9—C8—C14	120.9 (5)
N2—S1—C8	106.1 (2)	C9—C8—S1	119.3 (4)
C4—O1—H1	111 (5)	C14—C8—S1	119.7 (4)
C7—N1—N2	119.3 (4)	C8—C9—C10	118.9 (6)
N1—N2—S1	115.6 (3)	C8—C9—H9	120.6
N1—N2—H2A	115 (5)	C10—C9—H9	120.6
S1—N2—H2A	120 (5)	C9—C10—C11	121.5 (6)
C2—C1—C6	120.9 (5)	C9—C10—H10	119.2
C2—C1—I1	119.2 (4)	C11—C10—H10	119.2
C6—C1—I1	119.8 (4)	C13—C11—C10	117.8 (6)
C1—C2—C3	119.7 (5)	C13—C11—C12	121.4 (7)
C1—C2—H2	120.1	C10—C11—C12	120.7 (7)
C3—C2—H2	120.1	C11—C12—H12A	109.5
C2—C3—C4	120.0 (5)	C11—C12—H12B	109.5
C2—C3—H3	120.0	H12A—C12—H12B	109.5
C4—C3—H3	120.0	C11—C12—H12C	109.5
O1—C4—C3	117.3 (5)	H12A—C12—H12C	109.5
O1—C4—C5	121.9 (4)	H12B—C12—H12C	109.5
C3—C4—C5	120.8 (5)	C14—C13—C11	122.1 (6)
C6—C5—C4	118.3 (4)	C14—C13—H13	118.9
C6—C5—C7	119.8 (5)	C11—C13—H13	118.9
C4—C5—C7	122.0 (4)	C13—C14—C8	118.7 (6)
C1—C6—C5	120.3 (5)	C13—C14—H14	120.6
C1—C6—H6	119.9	C8—C14—H14	120.6
			
C7—N1—N2—S1	−162.9 (4)	C6—C5—C7—N1	−174.8 (5)
O2—S1—N2—N1	−36.8 (5)	C4—C5—C7—N1	6.4 (7)
O3—S1—N2—N1	−166.7 (4)	O2—S1—C8—C9	−153.8 (4)
C8—S1—N2—N1	78.8 (4)	O3—S1—C8—C9	−19.8 (5)
C6—C1—C2—C3	−1.7 (8)	N2—S1—C8—C9	92.0 (4)
I1—C1—C2—C3	178.6 (4)	O2—S1—C8—C14	29.4 (5)
C1—C2—C3—C4	1.9 (8)	O3—S1—C8—C14	163.4 (4)
C2—C3—C4—O1	179.3 (5)	N2—S1—C8—C14	−84.8 (5)
C2—C3—C4—C5	−1.1 (8)	C14—C8—C9—C10	−1.5 (8)
O1—C4—C5—C6	179.7 (5)	S1—C8—C9—C10	−178.3 (4)
C3—C4—C5—C6	0.1 (7)	C8—C9—C10—C11	0.1 (8)
O1—C4—C5—C7	−1.5 (7)	C9—C10—C11—C13	0.9 (9)
C3—C4—C5—C7	179.0 (5)	C9—C10—C11—C12	178.4 (6)
C2—C1—C6—C5	0.7 (8)	C10—C11—C13—C14	−0.5 (9)
I1—C1—C6—C5	−179.6 (4)	C12—C11—C13—C14	−177.9 (7)
C4—C5—C6—C1	0.1 (7)	C11—C13—C14—C8	−0.9 (10)
C7—C5—C6—C1	−178.8 (5)	C9—C8—C14—C13	1.9 (8)
N2—N1—C7—C5	−173.7 (4)	S1—C8—C14—C13	178.7 (5)

### Hydrogen-bond geometry (Å, º)

**Table:**

*D*—H···*A*	*D*—H	H···*A*	*D*···*A*	*D*—H···*A*
O1—H1···N1	0.82 (2)	1.91 (5)	2.589 (6)	139 (7)
N2—H2*A*···O1^i^	0.86 (2)	2.05 (2)	2.914 (6)	173 (6)

Symmetry code: (i) −*x*, *y*+1/2, −*z*.
